# What makes an orthopaedic paper highly citable? A bibliometric analysis of top orthopeadic journals with 10-year follow up

**DOI:** 10.1186/s40634-023-00631-x

**Published:** 2023-08-04

**Authors:** Mirjam Lutter, Henrik Rudolf, Robert Lenz, Thilo Hotfiel, Thomas Tischer

**Affiliations:** 1 Department of Orthopaedic Surgery, University Medical Center Rostock, Rostock, Germany; 2 Institute for Biostatistics and Informatics in Medicine and Ageing Research, University Medical Center Rostock, Rostock, Germany; 3 Center for Musculoskeletal Surgery Osnabrück (OZMC), Klinikum Osnabrück, Osnabrück, Germany; 4 Department of Orthopaedic and Trauma Surgery, Waldkrankenhaus, Erlangen, Germany

**Keywords:** Bibliometric analysis, Orthopaedic, Citations, Top paper, Impact factor

## Abstract

**Purpose:**

To examine a series of papers from top ranked orthopaedic journals with respect to the number of citations over a 10-year observation period to identify factors that lead to high citation rates.

**Methods:**

The Web of Science database was consulted to identify all published papers from the first-year term of 2010 (January-May) from four top orthopaedic journals: AJSM, Arthroscopy, JBJS Am and KSSTA. The database was used to analyze and compare the papers with respect to their characteristics and citations up to 2019. Basic information for each paper was collected including the author, country, study type and average citations per year (ACY). The most (Top20%) and least (Bottom20%) frequently cited papers were identified and differences were extracted.

**Results:**

Five hundred sixteen papers were included with a total of 19,261 citations. Most of the published papers were from the United States (*n* = 245). On average, a paper received 37.3 citations over the 10-year observation period. The most cited paper was cited 322 times. The most cited study type was randomized controlled trial (RCT) (Ø80.8). The Top20% papers were cited 37 times more often than the Bottom20%. Among the Top20%, the largest group was cohort study (*n* = 20) followed by case series (*n* = 19). Among others, the number of authors, the number of keywords and the number of references significantly correlated with the number of citations (*p* < 0.001).

**Conclusions:**

Factors influencing citation frequency were identified.

**Supplementary Information:**

The online version contains supplementary material available at 10.1186/s40634-023-00631-x.

## Introduction

It is a generally accepted goal of science to publish papers of the highest possible quality [36]. For authors, the impact factor (IF) of a journal is a significant contributing factor when deciding where to submit scientific work, since the perceived renown of a journal scales with its IF [24, 36, 37]. At the same time, journals advertise their IF and encourage authors to submit papers of the highest possible quality [36]. In addition, the current trend shows that researchers strive to publish a large quantity of papers [24]. This development has led to the assumption that the number of publications is the decisive criterion to measure the reputation of a scientist in professional circles [24]. According to findings, a high IF shapes the reward signal of scientists, at the prospect of publication [32]. Even if the high IF of a journal certifies a correspondingly high quality of a manuscript, however, it naturally does not allow any conclusions to be drawn about the quality of every article contained therein.

Currently, bibliometrics is being widely applied as a burgeoning method in numerous medical fields [3, 5, 15, 17, 19, 22, 23, 26, 27, 29–31, 40, 41, 44]. In recent years, bibliometric analyses have been used to find out which published paper was cited and how frequently [38–40]. Citation analyses can help to evaluate publications in terms of their quality of information to a particular field and to help researchers to better assess the published literature [1, 23, 26, 28]. For this purpose, for example, the Web of Science Core Collection (WOSCC) is recognized as one of the most suitable online databases for bibliometric analyses [33, 44]. Thus, how often a particular paper is cited by other authors is a measure of the scientific relevance of a paper [2, 25].

The aim of this study was to investigate which factors influence the citation frequency of orthopaedic papers and to determine which factors impact the citation rate. A further goal was to identify characteristics that distinguish the "top" orthopaedic papers. We hypothesized that studies with a high level of evidence such as randomized controlled trials or meta-analyses are cited most frequently.

Although there are already numerous studies that list and examine the most frequently papers on a particular medical specialty, to the best of our knowledge, no study exists that elaborates on the differences between well-cited papers and poorly-cited papers over a long period of time. The results could show authors which factors can be optimized to increase the likelihood of citations after publication.

## Methods

We examined papers from four major orthopaedic journals with high IF: the American Journal of Sports Medicine (AJSM, IF: 7.010), Arthrosocopy (IF: 5.973), The Journal of Bone and Joint Surgery American edition (JBJS Am, IF: 6.558) and Knee Surgery, Sports Traumatology Arthroscopy (KSSTA, IF: 4.114) (as of 2021). All of these are listed among the best 20 in Web of Science's Journal Citation Report in the category orthopaedics [11–14]. For comparison, the IFs of the journals in 2010 (time of publication of the studied articles) should also be mentioned here; AJSM (3,821), Arthroscopy (3,317), JBJS (2,969), KSSTA (1,857) [7–10] .

All consecutive papers published by the aforementioned journals during the first-year term from January 2010 to May 2010 were entered into a database. The JBJS was published twice per month and all others were published once per month. All papers, including case reports, commentaries, letters to the editor and editorials (*n* = 516), were considered and categorized. These papers were then individually analyzed for their characteristics and their citations were determined for every year using the Web of Science database over a total period of 10 years up to and including 2019 (*n* = 19,261 citations). Data collection was carried out by one author from December 2021 to January 2022; a sample check was performed by the second author; the last author provided appropriate quality assurance by cross-checking.

The following characteristics for each paper were collected via open access and subscription according to their availability: journal, first author, total number of authors, first author's country of origin, title, length of title in words, presence of a subtitle, wording of title, keywords and their number tables and figures, whether colored graphics were used, number of references, total citations up to and including 2019 and single citations per year from 2010 to 2019.

In order to make a possible statement about a statistical relationship between title and citation count, two sports orthopaedic surgeons (RL, TH) independently ranked the titles of the papers according to their subjective informative value on a scale of 1–10, for which 1 was definted as not appealing and 10 was defined as appealing by all measures.

Papers were then subclassified by authors according to study field, study type, field of research and study aim (Table 1). The categorization is based on a study classification published in the Lancet in 2002 [18]. We used quintiles regarding the total count of citations in the years 2010–2019 to compare the Top20%, Middle60% and Bottom20% papers.

**Table 1:** Classification of the papers

Study field	Study type	Field of research	Study aim
Clinical	Clinical Experimental: RCT	Upper Extremity: Shoulder	Description of new techniques or treatments
Basic Science	Clinical Experimental: non RCT	Upper Extremity: Elbow	Description of new injury pattern, disease, risk factors, etc
Reviews	Clinical Analytical: Cohort Study	Upper Extremity: Hand and Wrist	Evaluation of new techniques or treatments
Editorials/ Letter/Other	Clinical Analytical: Case Control Study	Lower Extremity: Hip	Review, Description of current state of the art, Guidelines
	Clinical Analytical: Cross Sectional	Lower Extremity: Knee	Validation studies (e.g., Classifications…)
	Clinical Descriptive: Case Report	Lower Extremity: Shin	Other
	Clinical Descriptive: Case Series	Lower Extremity: Ankle	
	Clinical: Technical notes (e.g., new surgical procedures)	Lower Extremity: Foot	
	Basic Science: Cell experimental	Spine, Pelvis, Trunk	
	Basic Science: Biomechanical study	Basis science (extra anatomical)	
	Basic Science: Animal study	General (epidemiological and others, extra anatomical)	
	Basic Science: Computer simulation		
	Review Clinical: Systematic Review		
	Review Clinical: Narrative Review		
	Review Clinical: Meta analysis		
	Review Basic Science: Systematic Review		
	Review Basic Science: Narrative Review		
	Review Basic Science: Meta analysis		
	Editorials/ Letter/ Other		

### Data analysis and statistical methods

Statistical analyses were performed using the statistical software package R version 4.1.2 and the packages *comparegroups* and *glm* [34]. As the first step, all papers (*n* = 516) were divided into a bottom (*n* = 108), middle (*n* = 305) and top range (*n* = 103) using quintiles according to the respective total citation numbers in the years 2010–2019 (Table 2). Regarding the subjective ranking, an inter-rater correlation calculation was performed. The Spearman R coefficient was adopted as the mathematical measure of correlation strength. We classified repeatability as very good (*R* > 0.75), good (*R* > 0.6), or poor to moderate (*R* < 0.59) [6]. Dependence of main categories of collected paper characteristics with top and bottom citation count was statistically tested by Chi-Square or Fisher’s exact test, applying a significance level of 0.05. Choice of chi-square test or Fishers exact test was according to expected cell counts. Mean and standard deviation were calculated for continuous variables.

**Table 2:** Part 1: Summary descriptives table by groups of citations. Summary descriptives table by groups of citations. Number of citations and percentages. Means and SD are presented

	**[ALL]**	**Bottom**	**Middle**	**Top**	**p overall**
	*N* = *516*	*N* = *108*	*N* = *305*	*N* = *103*	
**Citation count:**	37.3 (42.3)	2.78 (2.3)	27.2 (14.2)	103 (49.6)	< 0.001
**Journal:**					0.020
AJSM	128 (24.8%)	16 (14.8%)	76 (24.9%)	36 (35.0%)	
Arthroscopy	102 (19.8%)	26 (24.1%)	58 (19.0%)	18 (17.5%)	
JBJS	174 (33.7%)	45 (41.7%)	97 (31.8%)	32 (31.1%)	
KSSTA	112 (21.7%)	21 (19.4%)	74 (24.3%)	17 (16.5%)	
**Study field:**					< 0.001
Clinical	286 (55.4%)	45 (41.7%)	171 (56.1%)	70 (68.0%)	
Basic Science	110 (21.3%)	13 (12.0%)	85 (27.9%)	12 (11.7%)	
Reviews	60 (11.6%)	8 (7.4%)	32 (10.5%)	20 (19.4%)	
Editorials/Letter/Other	60 (11.6%)	42 (38.9%)	17 (5.6%)	1 (1.0%)	
**Study type:**					
Clinical Descriptive Case Series	100 (19.4%)	13 (12.0%)	68 (22.3%)	19 (18.4%)	
Clinical Analytical Case Control Study	31 (6.0%)	2 (1.9%)	21 (6.9%)	8 (7.8%)	
Clinical Analytical Cohort Study	45 (8.7%)	3 (2.8%)	22 (7.2%)	20 (19.4%)	
Clinical Analytical Cross Sectional	10 (1.9%)	0 (0.0%)	7 (2.3%)	3 (2.9%)	
Clinical Descriptive Case Report	41 (8.0%)	25 (23.1%)	16 (5.3%)	0 (0.0%)	
Clinical Experimental non RCT	20 (3.9%)	0 (0.0%)	14 (4.6%)	6 (5.8%)	
Clinical Experimental RCT	20 (3.9%)	0 (0.0%)	9 (3.0%)	11 (10.7%)	
Clinical Technical notes (e.g., new surgical procedures)	18 (3.5%)	2 (1.9%)	13 (4.3%)	3 (2.9%)	
Review Basic Science Narrative Review	13 (2.5%)	3 (2.8%)	5 (1.6%)	5 (4.9%)	
Review Clinical Metaanalysis	4 (0.8%)	0 (0.0%)	4 (1.3%)	0 (0.0%)	
Review Clinical Narrative Review	30 (5.8%)	5 (4.6%)	18 (5.9%)	7 (6.8%)	
Review Clinical Systematic Review	14 (2.7%)	0 (0.0%)	6 (2.0%)	8 (7.8%)	
Basic Science Animal study	19 (3.7%)	4 (3.7%)	12 (3.9%)	3 (2.9%)	
Basic Science Biomechanical study	73 (14.1%)	8 (7.4%)	60 (19.7%)	5 (4.9%)	
Basic Science Cell experimental	14 (2.7%)	1 (0.9%)	11 (3.6%)	2 (2.0%)	
Basic Science Computersimulation	4 (0.8%)	0 (0.0%)	2 (0.7%)	2 (1.9%)	
Editorials/ Letter/ Other	60 (11.6%)	42 (38.9%)	17 (5.6%)	1 (1.0%)	

In a second step, associations of collected journal characteristics with total citation count in 10 years was estimated by a multivariable generalized linear model with negative binomial distribution. Estimated coefficients of a predictor variable assess the difference in the logs of expected counts for each category in comparison to the reference, or for a one-unit change in the case of quantitative variables. The reference categories were as follows: in the field of journals: the *AJSM*; in the field of study type: *clinical descriptive: case series*; in the field of research: *knee*; and “*Description of new injury patterns, diseases, risk factories, *etc.” for the study aim (see Appendix Table A1).

## Results

### Analysis of the total collective

Overall, 516 papers with a total of 19.261 citations were examined. The average citation per paper was 37.3 (42.3 SD) and the most frequently cited paper was cited 322 times. The overall collective average citations per year (ACY) was 3.7 (5.1 SD) with 2015 being the year with the highest ACY (4.7; 5.5 SD). Otherwise, the average citation count per paper per year was nearly constant over the ten-year period after about two years after publication. The examined papers came from the JBJS Am (*n* = 174, 33.7%), AJSM (*n* = 128, 24.8%), KSSTA (*n* = 112, 21.7%) and Arthroscopy (*n* = 102, 19.8%). The AJSM had the highest average citation count per paper (50; 48.2 SD). Clinical studies were published most frequently (*n* = 286) Most publications originated from the USA (*n* = 245) All detailed results can be found in Tables 2, 3 and 4.

**Table 3:** Average citation per year per paper of the total collective

	ACY	SD	Max	Min
2010	0.8	1,4	12	0
2011	2,88	3,8	32	0
2012	4,1	5,0	37	0
2013	4,3	5,5	39	0
2014	3,9	4,8	36	0
2015	4,7	5,5	43	0
2016	4,4	5,7	41	0
2017	4,3	5,4	30	0
2018	4,1	5,5	40	0
2019	4,1	5,7	44	0
total	3,7	5,1	44	0

**Table 4:** Average citation per Journal

	AC	SD	Max	Min
AJSM	50	48,2	322	0
JBJS	35,3	38,6	224	0
KSSTA	33	46,3	250	0
Arthroscopy	29,7	32,0	150	0

### Study categories and their influence on citation

**Fig. 1:**
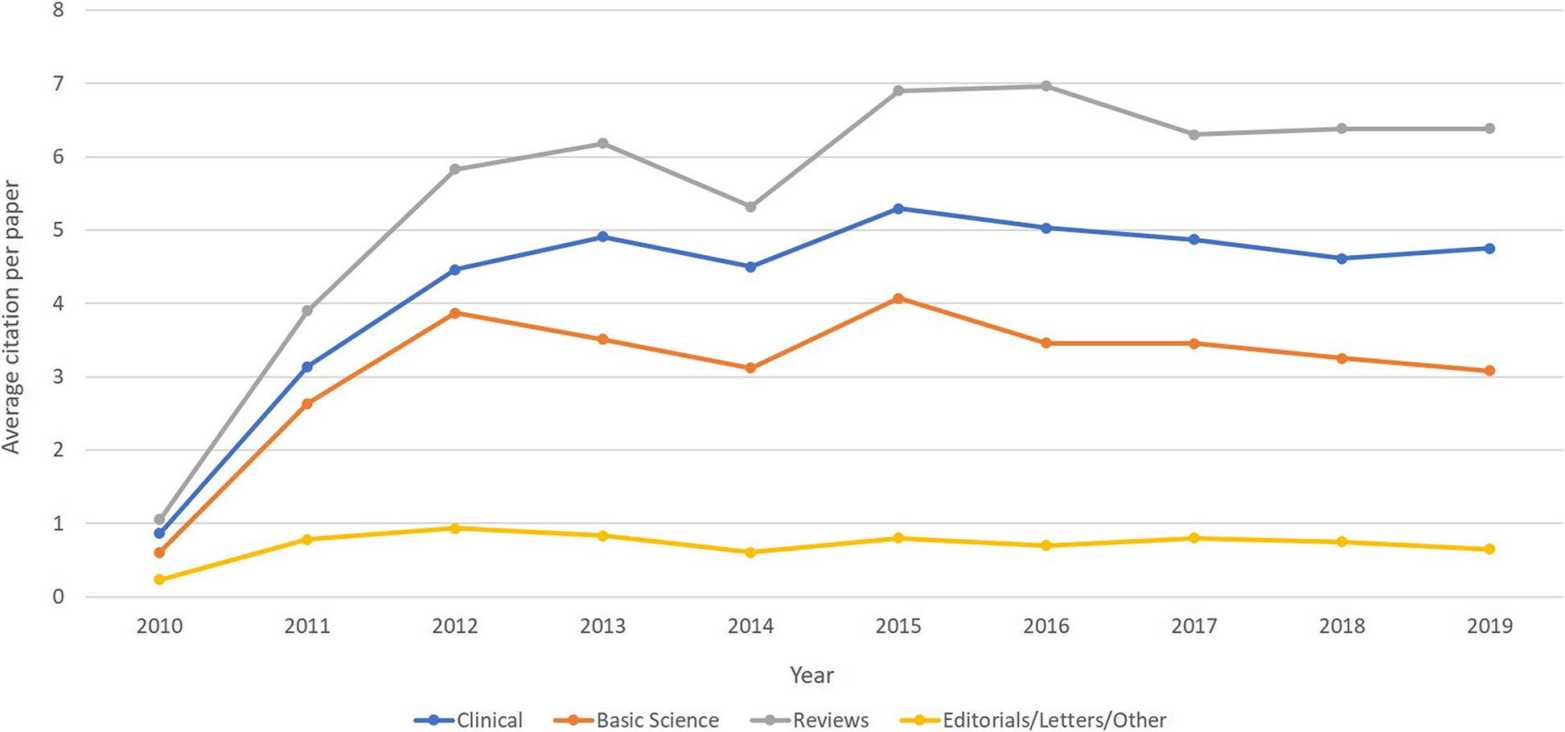
Average citation per paper per year of the total collective (according to study field)

Regarding the number of citations according to study fields, reviews displayed the highest average citations per paper (54.6; 56.7 SD) Regarding citation development over ten years, reviews were found to be the leading field of study (Fig. 1).

**Fig. 2:**
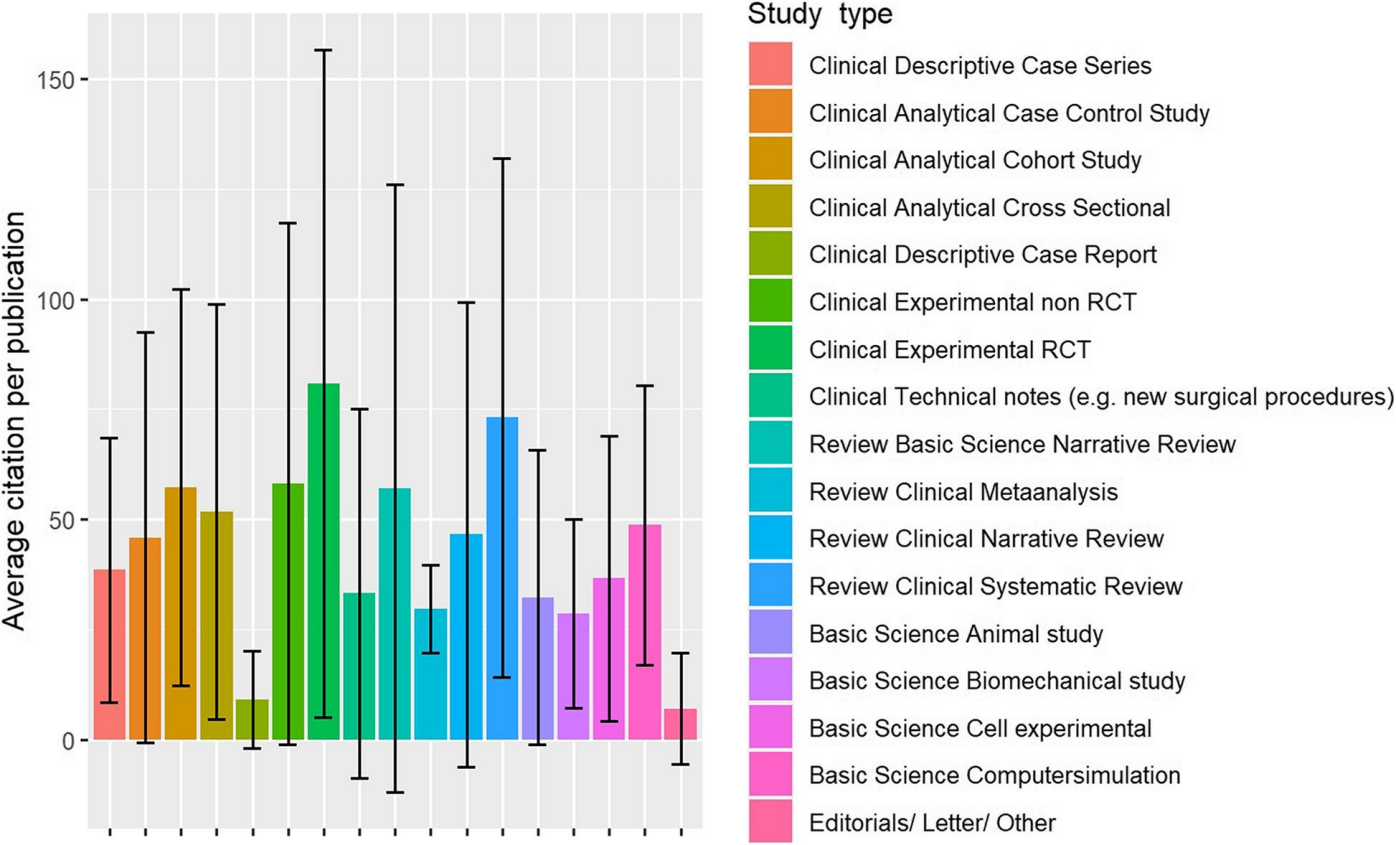
Average citation count per study type of the total collective as a bar plot. The bars symbolize the average citation frequency together with a measure of the standard deviation. As example Clinical Descriptive Case Report: The average citation rate is 9 with a standard deviation of 11

Among the study types, RCTs had the highest citation count (80.8, 75.7 SD) (Fig. 2).

The field of research had also an important influence on citation, with papers from basic science having the highest citation count (59.2; 62.4 SD) (Table 5). Concerning the field of research, basic science studies were cited significantly more frequently than studies concerning the knee (1.02, *p* = 0.018). In the category study aim, “Review, Description of current state of the art, Guidelines” had the highest citation count (51.6; 57.6 SD) (Table 6) However, the study aim was not shown to be a relevant factor in determining citation frequency. In terms of study type, experimental studies generate the most citations per year in the first few years after publication, but are caught up by review articles after about 6.5 years.

**Table 5:** Average citation per Field of research

	AC	SD	Max	Min
Upper Extremity: Shoulder	40,8	39,3	218	0
Upper Extremity: Elbow	41,9	79,2	322	0
Upper Extremity: Hand and Wrist	20,9	21,9	65	0
Lower Extremity: Knee	36,2	38,1	224	0
Lower Extremity: Shin	32,9	25,4	88	3
Lower Extremity: Ankle	41,8	43,9	232	1
Lower Extremity: Foot	22,9	27,2	101	1
Spine, Pelvis, Trunk	35,6	38,3	153	1
Basis science	59,2	62,4	250	0
General	14,7	20,6	88	0

**Table 6:** Average citation per study aim

	AC	SD	Max	Min
Description of new techniques or treatments	32,9	51,9	322	0
Description of new injury pattern, disease, risk factors, etc	35,5	38,4	218	0
Evaluation of new techniques or treatments	42,2	35,9	211	1
Review, Description of current state of the art, Guidlines	51,6	57,6	232	0
Validation studies	40	44,6	129	0
Other	9,3	15,6	74	0

Considering the dependence of the variables with the citation frequency, it was found that the study type has an especially strong influence on the citation frequency. Clinical analytical studies, such as RCT (1.49 times, *p* = 0.047) and non-RCT (1.83 times, *p* = 0.003), were cited more frequently than the reference case series, whereas biomechanical study (1.33 times, *p* = 0.032) and editorials/letter/other (2.3 times, *p* < 0.01) were cited less frequently. With regard to the subjective ranking of the titles by two sports orthopedists, interrater reliability was good with regard to the assessment of the manuscript titles. Therefore, the assessment was considered reliable. With regard to the other parameters surveyed, the number of authors (*p* < 0.001), the subjective evaluation of the title (*p* < 0.001), the title length (*p* = 0.029), the number of tables (*p* = 0.014) and the number of references (*p* < 0.001) significantly correlated with citation frequency; for each reference more in the paper, citations were 1.7% higher over ten years.

### Analysis of Top20% and Bottom20%

**Fig. 3:**
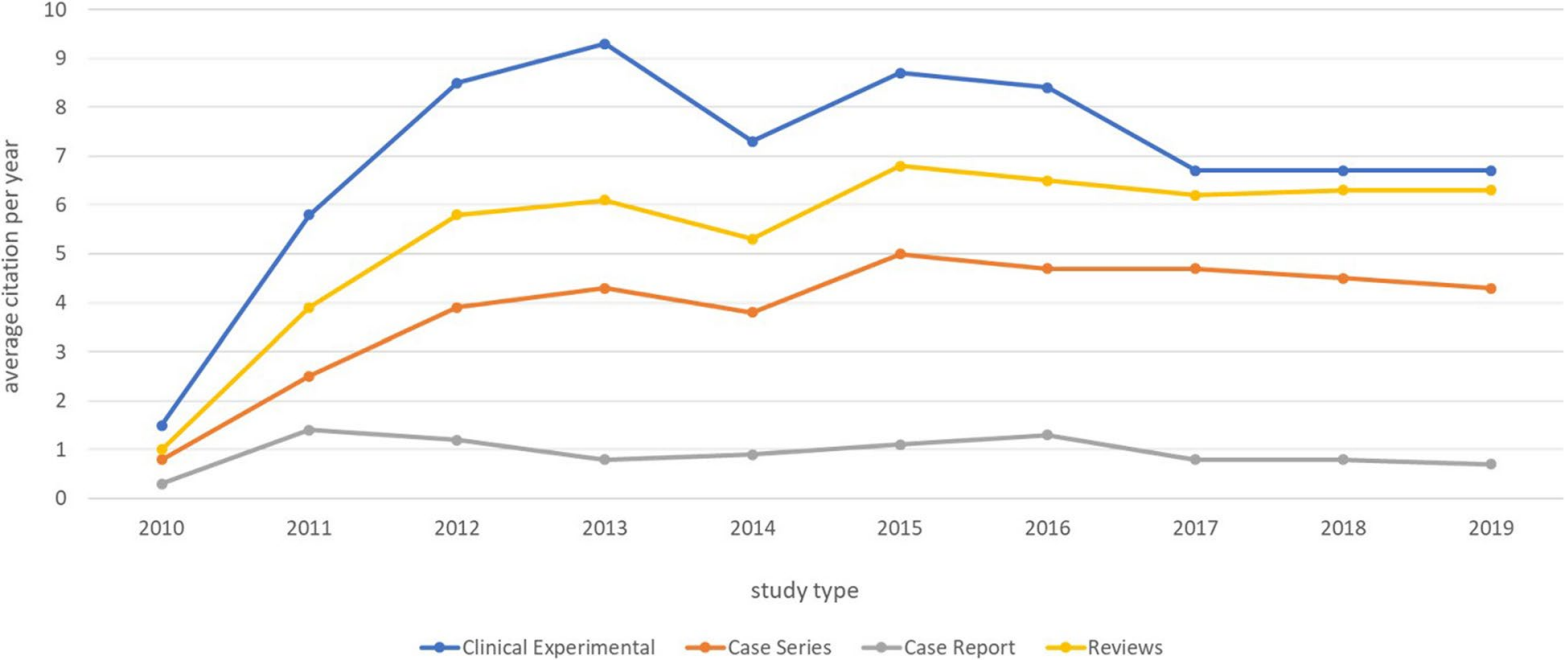
Average citation per year of selected categories (according to study type)

**Fig. 4:**
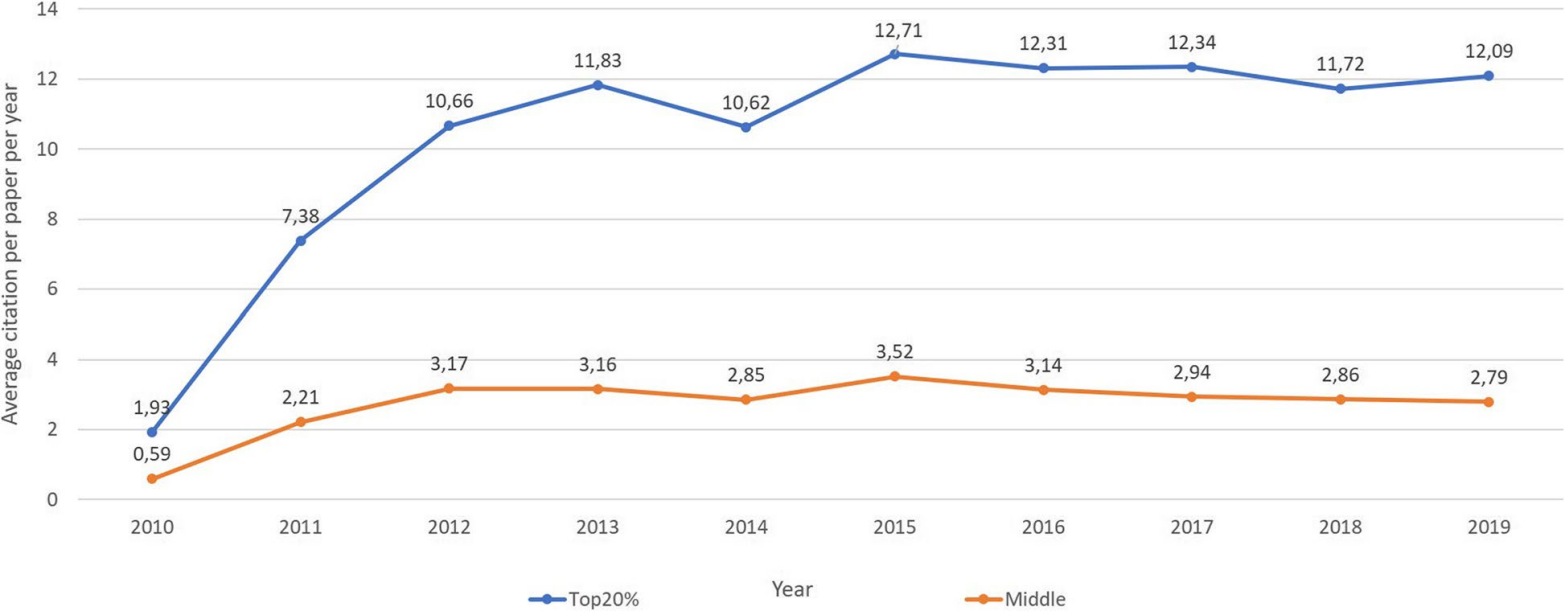
Average citation per paper per year for the Top20% and Middle60% in direct comparison

The average overall citation count of the Top20% (more than 60 citations) was 103 (49.6 SD) and the average overall citation count of the Bottom20% (7 or less citations) was 2.8 (2.3 SD), making the Top20% papers 37 times more frequently cited than the Bottom20% papers. The Top20% papers showed a largely constant citation trend after two years (Figs. 3 and 4).

The publishing journal had a significant effect on the distribution in the Top20%, Middle60% and Bottom20% papers (*p* = 0.02). Similarly, the study field was found to be a significant indicator for assessment in the Top20%, Middle60% and Bottom20% (*p* < 0.01). Reviews were more often among the Top20% (19.4% to 7.4% Bottom20%), whereas editorials/letters/other were found to have 38.9% in the Bottom20% and only 0.97% in the Top20%. In clinical trials, there was a clear tendency towards the Top20% instead of the Bottom20% (68% and 41.7%, respectively). The knee joint was by far the most frequently addressed field of research in both the Top20% and Bottom20% groups (35.9% and 29.6%, respectively) and could not be identified as a characteristic of either group. While most of the studies in the Top20% group focused on the study aim "Evaluation of new technique or treatments" (40.8%), the dominant study aim in the Bottom20% group was "Description of new injury pattern, disease, risk factors, etc." (37%). These showed a disproportionate distribution in favor of the respective categories.

Cohort Studies, RCTs and systematic reviews were disproportionately found in the Middle60% and Top20%, whereas case reports and editorials/letters/other were significantly more likely to be found in the Bottom20%. Biomechanical studies and case series showed no clear tendency regarding their distribution in either group. The most Top20% papers originated in the United States" (*n* = 51, 49.5%). Despite this fact, a comparison of the distribution in Top20%, Middle60% and Bottom20% showed that no country published a clearly higher proportion of Top20% papers in relation to the total number of papers. Details can be found in Table 2 Part 2 in the appendix. AJSM was most frequently represented in the Top20% (35%). In addition, the AJSM also showed the highest percentage of Top20% papers in terms of total number of papers (28%) and was the only journal to have a higher percentage of Top20% papers than that of the overall collective (35% compared to 24.8%). As a striking difference, the Top20% had significantly more references on average than the Bottom20% (39 and 16.9, respectively, *p* < 0.001). In addition, the Top20% were significantly characterized on average by a larger authorship (5.7 and 3.2, respectively, *p* < 0.001) or had a longer title in words on average (15.6 and 10.7, respectively). It was less relevant whether or not illustrations were printed in color. In the subjective ranking of titles by two sports orthopaedic surgeons, the Top20% were three rating points on average ahead of the Bottom20% (*p* < 0.001). Furthermore, it could be proven that Top20% papers show more tables (2.5 and 0.6, respectively, *p* < 0.001), more Figs. (4.0 and 2.7, respectively, *p* < 0.005) and more keywords (6.0 and 3.6, respectively, *p* < 0.001). Detailed results can be found in Table 2.

## Discussion

One main finding of this study is that the number of authors, title length, number of figures and reference quantity were statistically significant positive variables influencing the citation count. We found that larger authorship, longer titles, more numerous keywords, more tables and figures and a higher number of references positively correlated with the Top20% compared to the Bottom20%. Shekhani et al. were also able to show a positive correlation between the number of citations and authorship, the number of tables and figures and the number of references [37] in 2017. One possible reason is that a large author community or larger number of references generally indicates more complex research topics, which increases the value of the published paper. Additionally, a higher proportion of self-citations is likely with a larger authorship.

We also found that the Top20% papers showed a largely constant citation trend after two years that did not decline after ten years. Papers from the Middle60% did not show strong fluctuations with regard to citation frequency over the years, which is surprising because it would seem that in an ever more rapidly growing flood of publications, the importance of and general interest in individual papers declines rapidly over the years.

The most cited study field is reviews and the most cited study type is RCT. This conclusion was also reached by Khatra et al. in his study on the best-cited papers in sports medicine [23]. Considering the individual study types, experimental studies are ahead of reviews but are equaled by them after about 6.5 years. This can be explained by the fact that RCTs have a great impact on the current state of research due to their high level of evidence after a certain time, while reviews have a more general validity. Basic science was the best-cited field of research in the overall collective, with most papers in the Top20% addressing the knee joint. However, since the knee plays a central role in both the Bottom20% and Top20%, we could not statistically determine a preference for Top20% papers, but confirmed that it is the main topic of discussion across all groups. This underlines the importance of the knee joint in research circles of orthopaedics in this decade, a conclusion which was also reached by Khatra et al [23]. The study aim “Evaluation of new techniques or treatment” was the best cited on average, which is plausible, since an evaluated method is already somewhat established in certain circles and is of greater general interest than a completely unknown one. In the Top20% papers “evaluation of new techniques or treatment” makes up the largest part, whereas the primary study aim in the Bottom20% is “Description of new injury pattern, disease, or risk factors”. Evaluations and assessments of new findings are of general interest for the scientific community, whereas new findings are sometimes just of temporary interest.

We could not identify a unique geographic leader among the Top20%. Although almost half of the Top20% papers came from the USA half of all papers examined were of United States origin.Other authors, such as Khatra et al., state that the US has the largest research community with the most available funding available and that American authors are more likely to cite American work and publish in American journals [22, 23, 29]. However, Wu et al. highlighted the fact that China is exerting an ever-increasing influence on science and currently publishes the most research papers [42]. Although the US is one of the countries with the highest self-citation counts, [4, 43] the work of Bardeesi et al. concludes that these self-citations have little impact on the overall picture and therefore does not support the call by many scientists to exclude self-citations.

AJSM had the highest average citation count and the largest proportion of total papers in the Top20%, which is consistent with the fact that AJSM has the highest IF of the journals mentioned (7.010). It can also be said that even after a time interval of ten years, a high IF has a positive effect on the citation numbers.

In addition to the classic method of measuring the scientific influence of a paper by means of documented citations or the classification of a journal by means of an impact factor on databases such as Web of Science, alternative scores such as altmetrics are gaining influence by determining the digital attention of scientific content on the basis of clicks, comments, downloads, or mentions on social media platforms [16, 20, 21, 35]. The Altmetric Score generates the media attention of a publication from many sources, but its main flaw is obvious, as it is unclear whether the attention is positive or negative. Therefore, this is a parameter of the attention an article has received and not of scientific quality, although there can be a correlation between these parameters [16]. Hughes et al. concluded that sports and trauma orthopaedic journals with their own Twitter account have a higher IF than journals without, [20, 21] highlighting the importance of social media for scientific knowledge distribution. Buckarma et al. recognized social media as a suitable tool to disseminate scientific knowledge in a manuscript published in 2017 and already called on scientists to pay more attention to this topic. In a progressively digital and fast-paced world, such types of science analysis are expected to become increasingly influential and at least partially replace traditional established databases such as Web of Science.

Recent bibliometric analyses performed by others usually proceeded as following: the best cited papers at a defined time or period on a specific topic [22, 29, 40] or generally on the sports orthopaedic specialty were noted on one or a few days on large databases such as Scopus, Web of Science and PubMed and then examined in detail. Our approach was different, namely that we systematically compared papers published in early 2010 over a ten-year period, not only including the best cited papers. Including less well cited papers allows a more general statement about the characteristics of good papers and gives conclusions about the detailed citation development of a paper after publication for up to ten years.

This study is not without limitations: 1) self-citations by the authors were not excluded; 2) articles published in January 2010 have a time advantage and therefore a little more time to be quoted as compared to articles published in April 2010 and therefore may have a few more citations in 2010, however, over the ten-year period, this should only have a minimal impact; 3) the categories study field, study type, field of research and study aim are self-designed and extensive and lack universal reference standards; 4) the four journals examined were selected by consensus of the authors, so the statements in this paper do not necessarily reflect the entire field of sports orthopaedics; 5) the authors would like to emphasize once again that a high IF of a journal or a high number of citations of a publication is not the sole criterion for quality. However, in this study, a bibliometric understanding was used that articles with more citations are more likely to be considered top papers than those with few citations.

In summary, from our study results, the following recommendations can be made for highly cited publications:

1. more extensive authorships are advantageous
2. authors should aim for longer and therefore more concrete titles
3. specifying numerous keywords is favorable and suggests that the study can be found more easily
4. tables and figures are important because they are likely to make the study more understandable and descriptive
5. a broad repertoire of references should be considered, which allows the assumption of a sound basic understanding of the topic
6. RCTs and reviews are confirmed to be more frequently cited than other studies
7. basic science has a value in orthopaedics that should not be underestimated
8. studies that include an evaluation of a new technique or treatment are often cited by others

## Conclusion

The main results of this study are the statistical correlation between the number of citations with the number of authors, number of keywords and number of references, among others. In addition, we found that the Top20% papers showed a largely constant citation trend after two years for up to at least ten years. RCTs and reviews are cited most frequently.

### Supplementary Information

**Additional file 1: Appendix and Table 2 Part 2.**

## Data Availability

Data will be provided on request.
