# Supplementary material for: What makes an orthopaedic paper highly citable? A bibliometric analysis of top orthopeadic journals with 10-year follow up
Source: J Exp Orthop. 2023 Aug 4;10:78. doi: 10.1186/s40634-023-00631-x (PMC10403482; doi:10.1186/s40634-023-00631-x)
Supplement: Supplementary file 1 — Additional file 1: Appendix and Table 2 Part 2. [file 40634_2023_631_MOESM1_ESM.docx]

**Appendix**

**Table A1:** Generalized linear model

Evaluations: Influencing variables Number of citations

| **Observations** | 516 |
| --- | --- |
| **Dependent variable** | count_citations |
| **Type** | Generalized linear model |
| **Family** | Negative Binomial(1.6945) |
| **Link** | log |

| **χ²()** | 0.518 | 0.079 | 4489.313 | 4684.634 |
| --- | --- | --- | --- | --- |
| **Pseudo-R² (Cragg-Uhler)** | 0.518 | 0.079 | 4489.313 | 4684.634 |
| **Pseudo-R² (McFadden)** | 0.518 | 0.079 | 4489.313 | 4684.634 |
| **AIC** | 0.518 | 0.079 | 4489.313 | 4684.634 |
| **BIC** | 0.518 | 0.079 | 4489.313 | 4684.634 |

|  | **exp(Est.)** | **2.5%** | **97.5%** | **z val.** | **p** |
| --- | --- | --- | --- | --- | --- |
| **(Intercept)** | 4.340 | 2.386 | 7.892 | 4.811 | 0.000 |
| **JournalArthroscopy** | 1.050 | 0.808 | 1.364 | 0.367 | 0.714 |
| **JournalJBJS** | 1.018 | 0.799 | 1.297 | 0.147 | 0.883 |
| **JournalKSSTA** | 0.671 | 0.529 | 0.851 | -3.283 | 0.001 |
| **Study type Clinical Analytical Case Control Study** | 1.176 | 0.837 | 1.652 | 0.933 | 0.351 |
| **Study type Clinical Analytical Cohort Study** | 1.169 | 0.874 | 1.564 | 1.055 | 0.292 |
| **Study type Clinical Analytical Cross Sectional** | 0.919 | 0.533 | 1.583 | -0.305 | 0.761 |
| **Study type Clinical Descriptive Case Report** | 0.488 | 0.342 | 0.696 | -3.953 | 0.000 |
| **Study type Clinical Experimental non RCT** | 1.827 | 1.233 | 2.706 | 3.005 | 0.003 |
| **Study typeClinical Experimental RCT** | 1.491 | 1.005 | 2.212 | 1.985 | 0.047 |
| **Study type Clinical Technical notes (e.g. new surgical procedures)** | 0.921 | 0.583 | 1.455 | -0.354 | 0.723 |
| **Study type Review Basic Science Narrative Review** | 0.844 | 0.495 | 1.439 | -0.624 | 0.533 |
| **Study type Review Clinical Metaanalysis** | 0.577 | 0.248 | 1.341 | -1.278 | 0.201 |
| **Study type Review Clinical Narrative Review** | 0.644 | 0.417 | 0.994 | -1.988 | 0.047 |
| **Study type Review Clinical Systematic Review** | 1.272 | 0.758 | 2.134 | 0.910 | 0.363 |
| **Study type Basic Science Animal study** | 0.614 | 0.389 | 0.967 | -2.102 | 0.036 |
| **Study type Basic Science Biomechanical study** | 0.754 | 0.583 | 0.976 | -2.143 | 0.032 |
| **Study type Basic Science Cell experimental** | 0.829 | 0.501 | 1.371 | -0.730 | 0.465 |
| **Study type Basic Science Computersimulation** | 1.712 | 0.757 | 3.872 | 1.291 | 0.197 |
| **Study type Editorials/ Letter/ Other** | 0.265 | 0.165 | 0.424 | -5.529 | 0.000 |
| **Field of Research Lower Extremity Ankle** | 1.375 | 1.026 | 1.841 | 2.134 | 0.033 |
| **Field of Research Lower Extremity Foot** | 0.829 | 0.534 | 1.288 | -0.834 | 0.404 |
| **Field of Research Lower Extremity Hip** | 1.345 | 1.008 | 1.794 | 2.015 | 0.044 |
| **Field of Research Lower Extremity Shin** | 1.134 | 0.651 | 1.975 | 0.443 | 0.658 |
| **Field of Research Upper Extremity Elbow** | 0.956 | 0.615 | 1.487 | -0.198 | 0.843 |
| **Field of Research Upper Extremity Hand and Wrist** | 0.846 | 0.502 | 1.425 | -0.629 | 0.529 |
| **Field of Research Upper Extremity Shoulder** | 1.136 | 0.907 | 1.422 | 1.111 | 0.267 |
| **Field of Research Basis science (extra anatomical)** | 1.474 | 1.070 | 2.030 | 2.372 | 0.018 |
| **Field of Research General (epidemiological and others, extra anatomical)** | 1.265 | 0.856 | 1.869 | 1.178 | 0.239 |
| **Field of Research Spine, Pelvis, Trunk** | 1.584 | 1.089 | 2.305 | 2.405 | 0.016 |
| **Study aim Description of new techniques or treatments** | 1.098 | 0.851 | 1.416 | 0.720 | 0.472 |
| **Study aim Evaluation of new techniques or treatments** | 0.954 | 0.784 | 1.163 | -0.463 | 0.643 |
| **Study aim Other** | 1.404 | 0.857 | 2.300 | 1.348 | 0.178 |
| **Study aim Review, Description of current state of the art, Guidlines** | 1.181 | 0.835 | 1.671 | 0.942 | 0.346 |
| **Study aimValidation studies (e.g. Classifications,…)** | 1.153 | 0.613 | 2.167 | 0.441 | 0.659 |
| **Count authors** | 1.069 | 1.032 | 1.107 | 3.759 | 0.000 |
| **Title perception** | 1.204 | 1.133 | 1.279 | 6.017 | 0.000 |
| **Title length** | 1.018 | 1.002 | 1.034 | 2.179 | 0.029 |
| **Title typeFrage** | 0.821 | 0.527 | 1.278 | -0.874 | 0.382 |
| **Title typeoffen** | 1.064 | 0.774 | 1.463 | 0.381 | 0.703 |
| **Count keywords** | 0.966 | 0.932 | 1.001 | -1.886 | 0.059 |
| **Number of_tables** | 1.060 | 1.012 | 1.110 | 2.455 | 0.014 |
| **Number of_figures** | 1.020 | 0.989 | 1.051 | 1.247 | 0.212 |
| **Figures colornein** | 0.865 | 0.728 | 1.028 | -1.646 | 0.100 |
| **Numbers of References** | 1.018 | 1.012 | 1.023 | 6.378 | 0.000 |
| Standard errors: MLE |  |  |  |  |  |

**Table 2 Part 2:** Summary descriptives table by groups of citations. Number of citations and percentages

|  | **[ALL]** | **Bottom** | **Middle** | **Top** | **p overall** |
| --- | --- | --- | --- | --- | --- |
| **Field of research:** | | | | | |
| Lower Extremity Knee | 190(36.8%) | 32 (29.6%) | 121 (39.7%) | 37 (35.9%) |  |
| Lower Extremity Ankle | 41 (8.0%) | 5 (4.6%) | 24 (7.9%) | 12 (11.7%) |  |
| Lower Extremity Foot | 16 (3.1%) | 6 (5.6%) | 9 (3.0%) | 1 (1.0%) |  |
| Lower Extremity Hip | 46 (8.9%) | 11 (10.2%) | 25 (8.2%) | 10 (9.7%) |  |
| Lower Extremity Shin | 9 (1.7%) | 1 (0.9%) | 7 (2.3%) | 1 (1.0%) |  |
| Upper Extremity Elbow | 15 (2.9%) | 2 (1.9%) | 11 (3.6%) | 2 (1.9%) |  |
| Upper Extremity Hand and Wrist | 12 (2.3%) | 5 (4.6%) | 6 (2.0%) | 1 (1.0%) |  |
| Upper Extremity Shoulder | 83 (16.1%) | 13 (12.0%) | 49 (16.1%) | 21 (20.4%) |  |
| Basis science (extra anatomical) | 41 (8.0%) | 7 (6.5%) | 21 (7.0%) | 13 (12.6%) |  |
| General (epidemiological and others, extra anatomical) | 38 (7.4%) | 19 (17.6%) | 17 (5.6%) | 2 (1.9%) |  |
| Spine, Pelvis, Trunk | 25 (4.8%) | 7 (6.5%) | 15 (4.9%) | 3 (2.9%) |  |
| **Study aim:** | | | | | |
| Description of new injury pattern, disease, risk factors, etc. | 168 (32.6%) | 40 (37.0%) | 98 (32.1%) | 30 (29.1%) |  |
| Description of new techniques or treatments | 67 (13.0%) | 14 (13.0%) | 45 (14.8%) | 8 (7.8%) |  |
| Evaluation of new techniques or treatments | 168 (32.6%) | 14 (13.0%) | 112 (36.7%) | 42 (40.8%) |  |
| Other | 41 (8.0%) | 28 (25.9%) | 12 (3.9%) | 1 (1.0%) |  |
| Review, Description of current state of the art, Guidelines | 64 (12.4%) | 11 (10.2%) | 34 (11.1%) | 19 (18.4%) |  |
| Validation studies (e.g., Classifications…) | 8 (1.6%) | 1 (0.9%) | 4 (1.3%) | 3 (2.9%) |  |
| **Author count** | 4.7 (2.4) | 3.2 (2.1) | 4.9 (2.0) | 5.7 (2.9) | <0.001 |
| **First author country:** | | | | | |
| Egypt | 1 (0.2%) | 1 (0.9%) | 0 (0.0%) | 0 (0.0%) |  |
| Argentina | 1 (0.2%) | 0 (0.0%) | 1 (0.3%) | 0 (0.0%) |  |
| Australia | 8 (1.6%) | 0 (0.0%) | 6 (2.0%) | 2 (1.9%) |  |
| Belgium | 5 (1.0%) | 1 (0.9%) | 3 (1.0%) | 1 (1.0%) |  |
| Brazil | 1 (0.2%) | 0 (0.0%) | 1 (0.3%) | 0 (0.0%) |  |
| Chile | 2 (0.4%) | 1 (0.9%) | 0 (0.0%) | 1 (1.0%) |  |
| China | 17 (3.3%) | 2 (1.9%) | 13 (4.3%) | 2 (1.9%) |  |
| Denmark | 6 (1.2%) | 0 (0.0%) | 4 (1.3%) | 2 (1.9%) |  |
| Germany | 33 (6.4%) | 8 (7.4%) | 16 (5.3%) | 9 (8.7%) |  |
| Finland | 2 (0.4%) | 1 (0.9%) | 1 (0.3%) | 0 (0.0%) |  |
| France | 8 (1.6%) | 1 (0.9%) | 6 (2.0%) | 1 (1.0%) |  |
| Greece | 2 (0.4%) | 0 (0.0%) | 2 (0.7%) | 0 (0.0%) |  |
| India | 5 (1.0%) | 4 (3.7%) | 1 (0.3%) | 0 (0.0%) |  |
| Iran | 1 (0.2%) | 0 (0.0%) | 1 (0.3%) | 0 (0.0%) |  |
| Ireland | 1 (0.2%) | 0 (0.0%) | 1 (0.3%) | 0 (0.0%) |  |
| Israel | 2 (0.4%) | 1 (0.9%) | 1 (0.3%) | 0 (0.0%) |  |
| Italy | 12 (2.3%) | 2 (1.9%) | 7 (2.3%) | 3 (2.9%) |  |
| Japan | 25 (4.8%) | 4 (3.7%) | 19 (6.2%) | 2 (1.9%) |  |
| Canada | 16 (3.1%) | 6 (5.6%) | 10 (3.3%) | 0 (0.0%) |  |
| Luxembourg | 2 (0.4%) | 0 (0.0%) | 2 (0.7%) | 0 (0.0%) |  |
| Netherlands | 18 (3.5%) | 2 (1.9%) | 11 (3.6%) | 5 (4.9%) |  |
| Norway | 7 (1.4%) | 0 (0.0%) | 4 (1.3%) | 3 (2.9%) |  |
| Austria | 4 (0.8%) | 0 (0.0%) | 3 (1.0%) | 1 (1.0%) |  |
| Sweden | 9 (1.7%) | 3 (2.8%) | 3 (1.0%) | 3 (2.9%) |  |
| Switzerland | 11 (2.1%) | 1 (0.9%) | 5 (1.6%) | 5 (4.9%) |  |
| Singapore | 3 (0.6%) | 2 (1.9%) | 1 (0.3%) | 0 (0.0%) |  |
| Spain | 9 (1.7%) | 1 (0.9%) | 4 (1.3%) | 4 (3.9%) |  |
| South Korea | 32 (6.2%) | 5 (4.6%) | 22 (7.2%) | 5 (4.9%) |  |
| Taiwan | 5 (1.0%) | 2 (1.9%) | 3 (1.0%) | 0 (0.0%) |  |
| Turkey | 4 (0.8%) | 2 (1.9%) | 1 (0.3%) | 1 (1.0%) |  |
| UK | 19 (3.7%) | 7 (6.5%) | 10 (3.3%) | 2 (1.9%) |  |
| USA | 245 (47.5%) | 51 (47.2%) | 143 (46.9%) | 51 (49.5%) |  |
| **title perception** | 4.95 (1.7) | 3.42 (1.7) | 5.13 (1.5) | 6.03 (1.3) | <0.001 |
| **title length** | 13.6 (5.3) | 10.7 (5.4) | 13.9 (4.5) | 15.6 (5.9) | <0.001 |
| **title type:** |  |  |  |  | 0.015 |
| statement | 32 (6.2%) | 10 (9.3%) | 17 (5.6%) | 5 (4.9%) |  |
| question | 29 (5.6%) | 12 (11.1%) | 10 (3.3%) | 7 (6.8%) |  |
| open wording | 455 (88.2%) | 86 (79.6%) | 278 (91.1%) | 91 (88.3%) |  |
| **Keyword count** | 5.4 (3.0) | 3.6 (3.30) | 6.0 (2.8) | 6.0 (2.5) | <0.001 |
| **Number of tables** | 1.8 (1.9) | 0.6 (1.2) | 2.0 (1.8) | 2.5 (2.0) | <0.001 |
| **Number of figures** | 3.3 (2.9) | 2.8 (3.1) | 3.3 (2.5) | 4.0 (3.6) | 0.005 |
| **Color figures:** |  |  |  |  | 0.035 |
| yes | 253 (49.0%) | 41 (38.0%) | 159 (52.1%) | 53 (51.5%) |  |
| no | 263 (51.0%) | 67 (62.0%) | 146 (48.0%) | 50 (48.5%) |  |
| **number of references** | 30.4 (19.4) | 16.9 (15.5) | 32.2 (16.9) | 39.0 (22.6) | <0.001 |
